# Piezoelectric Nanogenerators Fabricated Using Spin Coating of Poly(vinylidene fluoride) and ZnO Composite

**DOI:** 10.3390/nano13071289

**Published:** 2023-04-06

**Authors:** Md. Jahirul Islam, Hyeji Lee, Kihak Lee, Chanseob Cho, Bonghwan Kim

**Affiliations:** 1 Department of Semiconductor Electronic Engineering, Daegu Catholic University, Gyeongsan 38430, Republic of Korea; jahirruet@cu.ac.kr (M.J.I.);; 2 School of Electronics Engineering, Kyungpook National University, Daegu 41566, Republic of Korea

**Keywords:** spin coating, piezoelectric, nanogenerator

## Abstract

In this context, the open-circuit voltage generated by either poly (vinylidene fluoride) or PVDF and ZnO composite sample before being enhanced to 4.2 V compared to 1.2 V for the samples of pure PVDF. The spin coating method was used to create a composite film, which served as a piezoelectric nanogenerator (PNG). Zinc oxide (ZnO) nanoparticles and PVDF serve as the matrix for the coating structure. Thin films were created that employed the spin coating method to achieve the desired results of ZnO’s non-brittle outcome and piezoelectric characteristics, as well as PVDF for use in self-powered devices. Scanning electron microscopy (SEM), X-ray diffraction (XRD), and fourier transform infrared (FT-IR) were used to evaluate the properties of these formations. The electrical properties of the film were measured using an oscilloscope. Results indicated that by adding ZnO nanoparticles to the PVDF samples, piezoelectric capabilities were enhanced compared to samples containing PVDF only. These results point to promising uses for various wearable devices, such as water strider robot systems and self-operating equipment.

## 1. Introduction

There are many small-scale energy sources available. Despite being negligible when seen individually, the sum of these energies can be large [1]. Sensors, defense technologies, and personal electronics all benefit from the usage of self-powering systems that can gather energy from the environment [2]. For compact and power-efficient devices, this is very crucial [3,4,5,6]. In a variety of applications, thermal, solar, and mechanical (such as wind, friction, and body movement) energies are frequently used as energy scavengers [7]. One of the most crucial materials for energy harvesting is a piezoelectric material, with numerous studies being conducted on this topic [8,9,10,11,12,13,14,15]. Due to the creation of an electric field in reaction to mechanical stress, piezoelectric materials are able to transform mechanical energy into electrical energy. In-depth research has been conducted on piezoelectric materials for a variety of uses, such as actuators, sensors, and energy harvesters. Piezoelectric nanogenerators (PENGs) have received a lot of interest lately because of their ability to capture environmental mechanical energy and transform it into electrical energy. PENGs can be used for a variety of purposes, including creating implanted medical devices, environmental monitoring, and powering portable electronic gadgets. PENGs have been tested using a variety of materials, including composites, polymers, and piezoelectric ceramics. Due to its superior piezoelectric capabilities, poly (vinylidene fluoride) (PVDF) is one of the most frequently utilized polymers for PENGs. Because to their high piezoelectric coefficients and low toxicity, zinc oxide (ZnO) nanoparticles have also been thoroughly researched for application in PENGs. Traditional piezoelectric materials, which are typically thin films, respond inefficiently to light mechanical forces [16]. Another class of piezoelectric materials that has demonstrated the capacity to convert mechanical energy from body movements, vibrations, or different forces into electrical energy are semi-conductive nanowires, such as ZnO, InN, GaN, CdS, and ZnS [17,18,19,20,21]. These materials could be a strong contender for adding self-powering capabilities to small electrical devices. However, this particular class of nanogenerators needs precise, well-organized manufacturing conditions, and they are extremely fragile and difficult to incorporate into soft materials, such as textiles or plastic films [22,23,24,25,26,27]. Polymeric fibers are significantly softer and more flexible than nanowires. They can withstand more mechanical deformation because they have higher levels of strain [1]. One of the most widely used piezoelectric polymers is polyvinylidene fluoride (PVDF), which is valued for its great flexibility, biocompatibility, and affordability. Because of these characteristics, PVDF is a good option for energy transformation applications, such as tiny electromechanical devices, actuators, and energy harvesters [28,29]. Numerous recent publications describe the fabrication of PVDF nanofibers using electrospinning as an energy harvesting device [30]. Even though the resulting nanogenerators only displayed very low piezoelectric outputs (up to 30 mV and 3 nA) [1,31], a single PVDF nanofiber has been strung over a few electrodes by a near-field electrospinning method to gather minor mechanical vibration. Another type of flexible device for converting mechanical to electrical energy is aligned nanofiber mats and randomly oriented electrospun PVDF nanofiber webs. The semi-crystalline polymer PVDF has four distinct crystal phases: α, β and δ. The non-polar α phase is typically present in melting form, polymer solutions, and commercially available films. Due to the unpredictable direction of the dipole moments in this phase, they cancel one another out. Dipole moments pointing in the same direction are said to be in the β phase; this phase is what gives PVDF its piezoelectric properties [28]. High β crystals should be produced in PVDF in order to obtain the requisite piezoelectric characteristics [1]. Mechanical elongating and an electrical poling treatment are often used to achieve the first goal [32]. For near-field spin coating PVDF nanofilms, extra poling treatment is not required [33]. The performance of the PVDF nanofibers has been studied extensively, though it still has a low output efficiency. In the majority of these studies, the introduction of nanoparticles, such as CNTs, Graphene, BaTiO_3_, and ZnO, has improved this performance [34,35,36,37,38,39,40]. ZnO is one of the possible options for enhancing the performance of nanogenerators due to its piezoelectric capabilities [41,42]. In this work, PENGs were created utilizing a composite material made of ZnO and PVDF nanoparticles. The spin coating method was utilized to deposit the composite film onto a flexible substrate, enabling the production of PENGs at a cheap cost and with excellent reproducibility. The primary goal of this study is to increase the output efficiency of a flexible piezoelectric nanogenerator (PNG) by spin coating a piezopolymer (PVDF) and a nanoparticle ZnO together. This goal was achieved by fabricating and analysing nano-composite films with various particle-to-matrix volume ratios.

## 2. Literature Review

In recent years, researchers have focused on optimizing the PVDF-ZnO PENG fabrication process to improve their performance. To avoid the need for post-poling treatment in hybrid structures, Fakhri et al. [43] developed a unique hybrid piezoelectric structure based on poly (vinylidene difluoride) nanofibers (PVDF NFs) and zinc oxide nanorods (ZnO NRs). This research also discusses the mechanisms that improve the hybrid structure’s electrical performance. Comparing the hybrid structure to pure ZnO NRs and PVDF NFs nanogenerators, it was discovered that the hybrid structure’s output power was increased. Nanogenerators were made using an electrospinning approach, and ZnO nanorods were grown using a hydrothermal process. With the hybrid construction, the open-circuit voltage was measured at 0.4 V.

By incorporating ZnO nanorods into the PVDF matrix, Singh et al. [44] demonstrated that the polar crystalline phase of PVDF, which is responsible for the piezoelectric ability, can be increased from 48.2% to 76.1%. Nanocomposite films have been used to create nanogenerators using the drop-casting process; it has been discovered that ZnO loading improves PVDF’s piezoresponse. With a 15 wt% ZnO-loaded PVDF nanocomposite film, a maximum open-circuit voltage of 1.81 V and a short-circuit current of 0.57 A are attained.

Zinc oxide (ZnO) nanowires and poly (vinylidene fluoride) (PVDF) polymer are used in a hybrid piezoelectric structure that Choi et al. [45] analyzed for power increases. Spin coating was used to create thin films. According to several analyses, the ZnO nanowires in the hybrid structure provided internal strain to the PVDF, increasing the electrical power output of a hybrid nanogenerator. Peak to peak measurement of the open-circuit voltage was 0.71 V.

In their study, Sabry et al. [46] reported that the electrospinning method was used to create fibers of polyvinylidene fluoride/zinc oxide (PVDF/ZnO) nanocomposites at different concentrations and shapes. The nanocomposites included PVDF matrices in varying concentrations and were shaped by synthesizing ZnO into varied forms. The output voltage for the constructed nanogenerator was measured at 3.6 V at 31.88 N of applied mechanical force.

Li et al. [47] reported, n-propylamine (PA), a dispersion, and silane coupling agent were both added concurrently to ZnO nanoparticles (NPs) to modify them. Moreover, spin coating was used to create PVDF-TrFE/modified ZnO composite films with excellent crystallinity and a large specific surface area. These films served as the piezoelectric nanogenerator’s active layer (PENG). The PENG’s output voltage remained at 2.40 V after 1000 testing cycles, showing that the generator had a good mechanical durability.

As piezoelectric materials, zinc oxide (ZnO) nanorods were created by Oo et al. [48] using the spray pyrolysis and hydrothermal methods, respectively. After that, 500 rpm spin coating of poly (vinylidene difluoride, or PVDF) was performed on ZnO nanorods. The ZnO film, pure PVDF film, and ZnO-PVDF were then put together. The composite ZnO-PVDF film had higher PFM amplitudes and greater output voltage from mechanical tapping as compared to the ZnO and pure PVDF films. The ZnO-PVDF was able to output 28 mV.

ZnO nanoparticle-PVDF composite thin films with spin coating were produced and described by Jin et al. [49], who also reported on different metal doped ZnO nanostructures. On the basis of these piezoelectric composite thin films, a number of flexible piezoelectric nanogenerators (PENGs) were developed. Individual studies and comparisons of the voltage output from samples of cobalt (Co), sodium (Na), silver (Ag), and lithium (Li) doped ZnO-PVDF composite and pure ZnO-PVDF were conducted. The Li-ZnO-based device generated the highest peak-to-peak voltage (3.43 Vpp) under the identical experimental circumstances; however, Co-ZnO, Na-ZnO, and Ag-ZnO are 1.2, 4.9, and 5.4 times larger, respectively.

**Figure 1:**
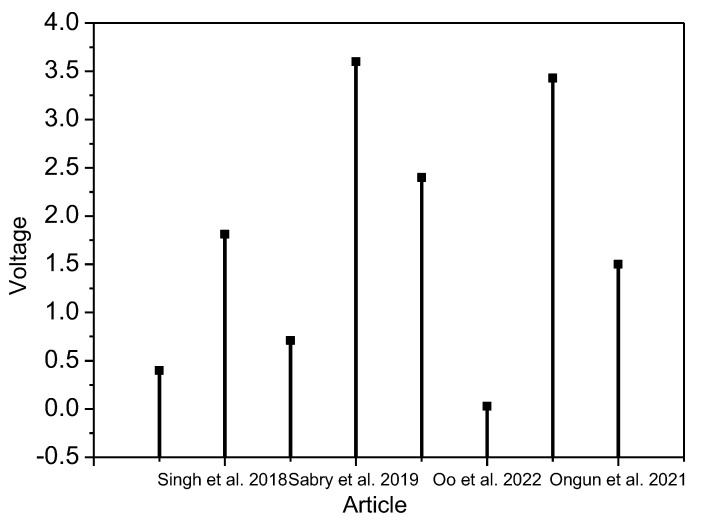
Output voltage measured in different articles in literature review [44,46,48,50].

In another study, Ongun et al. [50] showed how silver-doped zinc oxide (ZnO@Ag) nanoparticles (NPs) were used to increase the piezoelectric content of electrospun-phase poly (vinylidene fluoride) PVDF nanofibers by synthesizing nanoscale zinc oxide (ZnO) with varied concentrations (1, 3, and 5 wt%). Nanofibers were created using the electrospinning technique. All the synthesized materials’ structural and morphological characteristics were examined. Compared to undoped PVDF samples, the electrical output of 5 wt% Ag-doped ZnO-based -PVDF nanofibers rose from 0.5 to 1.5 V. In Figure 1, we analyze the above-mentioned papers and present their output voltage measured at different mechanical force levels.

## 3. Experimental Section

### 3.1. Materials

Firstly, Acetone (Daejung Co., Busan, Republic of Korea) and N, N-Dimethylformamide (DMF, Daejung Co., Republic of Korea) were employed as the solvents, while Poly (vinylidene fluoride) pellets (Mw 180,000 by GPC) were purchased from Sigma Aldrich Co. (St. Louis, MO, USA) ZnO nano-powder was purchased from Duskan pure chemicals, Korea, with an average particle size of about 15 nm.

### 3.2. Selection of PVDF-ZnO for Fabrication of Piezoelectric Nanogenerator

PVDF-ZnO was a preferred choice for piezoelectric nanogenerators due to its unique features and benefits over other materials. The following are some justifications for choosing PVDF-ZnO for piezoelectric nanogenerators:

High piezoelectric coefficient: Since PVDF has a high piezoelectric coefficient, it can produce a lot of electrical energy from only a little mechanical energy. PVDF’s piezoelectric capabilities were further improved by adding ZnO, leading to a better energy conversion efficiency.

- Flexibility: PVDF is a flexible polymer, making it appropriate for application in flexible and wearable technology. The flexibility of the material was not considerably impacted by the inclusion of ZnO nanoparticles.
- Biocompatibility: PVDF may be employed in biomedical applications without endangering live tissues since it is biocompatible. This qualified PVDF-ZnO as a material that could be used in biological applications, including biosensors and implanted medical devices.
- Easy to fabricate: PVDF-ZnO may be made utilizing a number of different methods, including as electrospinning, solution casting, and spin coating.
- Wide bandgap: ZnO has a large bandgap, allowing it to function in severe conditions and at high temperatures.

### 3.3. Doping of ZnO in PVDF

To improve the piezoelectric characteristics of polyvinylidene fluoride (PVDF), zinc oxide (ZnO) was doped into the material. The formation of a heterojunction between the two materials, which resulted in a synergistic influence on their piezoelectric characteristics, was the mechanism of ZnO doping in PVDF. ZnO nanoparticles were incorporated into the PVDF matrix during the doping process. Due to the ZnO nanoparticles’ polarity, there was a polarization mismatch between them and the PVDF matrix. As a result, a heterojunction formed at the interface of the two materials, improving their piezoelectric capabilities. The crystal structure of the material deformed in reaction to an external force, producing an electrical charge; this is what gave rise to the piezoelectric effect. When PVDF-ZnO was involved, an external force deformed the PVDF matrix, causing the ZnO nanoparticles to move. A dipole moment was produced by this displacement, which in turn caused an electric field and an electrical charge. The addition of ZnO to PVDF also made the material’s matrix more crystallin, which improved its piezoelectric characteristics. This occurred because highly crystalline materials had the largest piezoelectric effect. Overall, the heterojunction that formed between the two materials as a result of ZnO doping in PVDF had a synergistic influence on the materials’ piezoelectric capabilities.

### 3.4. Fabrication of PVDF and PVDF/ZnO Nanocomposites and PNGs

**Figure 2:**
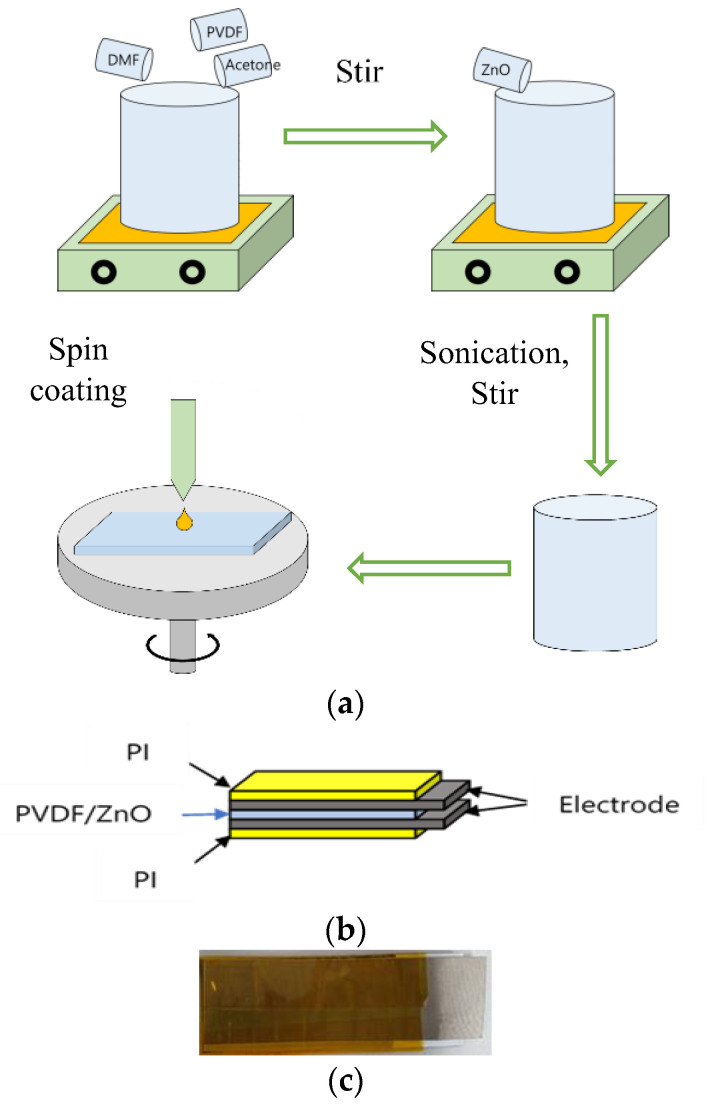
(**a**) Composite and film preparation process, (**b**) PNG design, (**c**) fabricated PNG.

As in Figure 2, to create a PVDF solution, PVDF pellets were added to a solvent mixture of DMF and acetone (DMF: Acetone = 6 wt.: 4 wt.); we then magnetically stirred the resulting heterogeneous solution at 60 °C and 800 rpm for almost three hours. A transparent homogeneous solution was prepared. In the mixture, 26 wt.% of PVDF was used. Secondly, the ZnO nanopowder was added to the produced PVDF solution at the proper concentration (Table 1) and the mixture was sonicated for 20 min (Cycle: 0/5, Amplitude: 60). The solution was then magnetically stirred at 60 °C and 800 rpm for five hours. Finally, a white homogeneous solution was produced that was ready for spin coating. The solution was held in a vacuum chamber after preparation to remove the bubble formed during mixing. The ACE-200 spin coater was loaded with sliding glass. The 2 mL of solution was put over the sliding glass, which was subsequently spin coated (at 700 rpm, 3.0 ramp angle, and time 60 s). At 60 °C, the coated glass was cured. A thin film was, finally, prepared to function as PNG. Lesser ZnO results in poorer electrical output, whereas more ZnO—more than 15wt.%—causes the film to become brittle, making sample SN-8 the best choice.

### 3.5. Characterization

X-ray diffraction (XRD, DIATOME, MPD for thin film) was used to determine the crystalline phases and XRD patterns of all samples. Fourier transform infrared spectroscopy (FT-IR) spectra was were captured using a spectrometer (PERKIN ELMER, Spectrum100, Kwun Tong, Hong Kong). The surface morphologies of the composite were observed using a scanning electron microscope (SEM, Tescan, Mira III, Brno, Czech Republic). Using a 200 MHz oscilloscope (Wavesurfer 3024z, Teledyne LeCroy, Chestnut Ridge, NY, USA), while the open-circuit voltages of PNGs were measured.

### 3.6. Working of Piezoelectric Sensor

**Figure 3:**
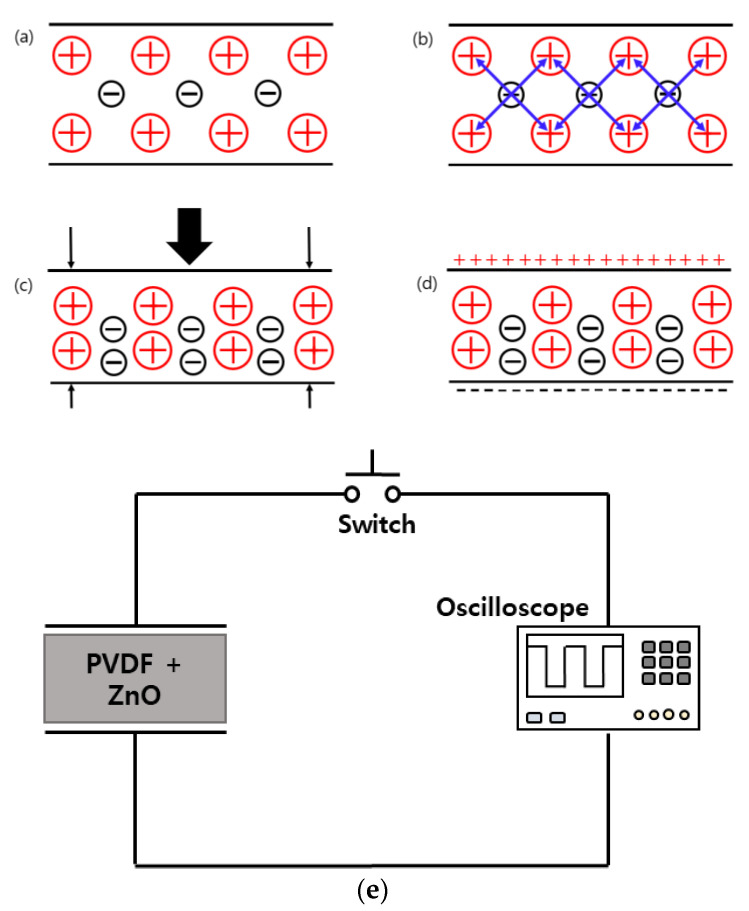
(**a**–**d**) Working principal of piezoelectric sensor, (**e**) electrical setup of nanogenerator with oscilloscope.

The lead zirconate titanate (PZT) material generates an identical amount of electrical charge across its crystal faces when pressure or acceleration are applied. The relationship between electrical charge and applied pressure is linear. Static pressure cannot be measured with a piezoelectric sensor. Piezoelectric working is summarized in Figure 3a–d. The output signal will be 0 at constant pressure. The charges in a piezoelectric crystal are perfectly balanced even in an asymmetrical arrangement. Since the effects of the charges cancel each other out, the crystal faces do not have any net charge. The crystal’s charge falls out of equilibrium when it is compressed. As a result, from this point on, the effects of charge do not cancel one another, causing net positive and negative charge to emerge on the opposing faces of the crystal. Therefore, piezoelectricity is the process of producing voltage across the opposing face of a crystal by applying pressure on it.

### 3.7. Electrical Characterization

The electrical characterization of a PVDF-ZnO piezoelectric nanogenerator involved measuring the open-circuit voltage generated by the device in response to an external mechanical force.

The experimental setup worked as follows: (1) a PVDF film was coated with a combination of ZnO nanoparticles utilizing spin coating processes to create the PVDF-ZnO nanogenerator; (2) the nanogenerator was fastened to a hitting machine and tiny iron rods struck it with a mechanical force provided by a dc motor; and (3) a 12 V laboratory dc power source powered the dc motor. Moreover, while the nanogenerator was on the table and being pressed by hand, the voltage generated from it was monitored. An oscilloscope with a high impedance (100 MΩ) voltage probe was used to measure the device’s electrical output by connecting two opposed electrodes to the oscilloscope’s opposite terminal (see Figure 3e).

The extraction of open-circuit voltage occurred as follows: (1) a mechanical force was applied to the nanogenerator, causing it to produce an electrical charge; (2) the charge built on the electrodes of the gadget, causing a potential difference between them; and (3) the device’s open-circuit voltage, or the voltage produced when there is no load attached to the device, was then measured using the oscilloscope. To achieve an average value and guarantee that the results were repeatable, the open-circuit voltage was usually tested numerous times. To thoroughly assess the performance of a PVDF-ZnO piezoelectric nanogenerator, measurements of short-circuit current, power output, and energy conversion efficiency may also be made in addition to open-circuit voltage. Similar experimental configurations and measurement methods to those previously mentioned can be used to derive these parameters.

## 4. Result and Discussion

### 4.1. Scanning Electron Microscope (SEM)

**Figure 4:**
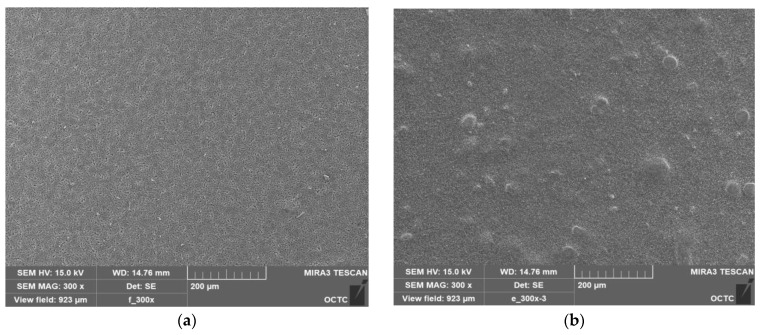

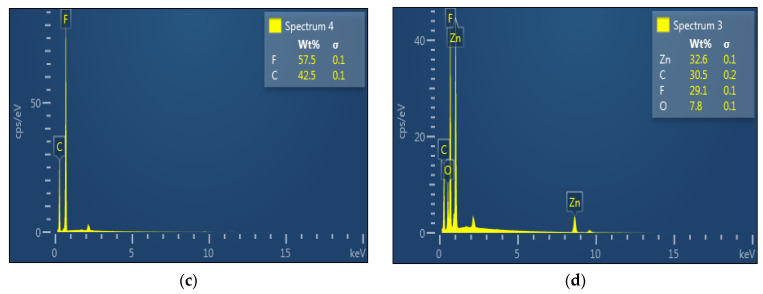
SEM images (**a**) PVDF, (**b**) PVDF/ZnO; corresponding EDS spectrum (**c**) PVDF, (**d**) PVDF/ZnO.

A concentrated electron beam is used in scanning electron microscopy (SEM), a form of microscopy, to produce high-resolution pictures of a sample’s surface. The interaction between the electrons and the sample surface results in signals that may be picked up and used to create a picture. SEM is a potent surface analysis instrument that may offer comprehensive details on the shape and composition of a variety of materials. The steps in our SEM study are as follows: (1) to increase the sample’s conductivity and avoid charging during imaging, a small layer of conductive material, such as gold, was applied to it; (2) the sample is inserted in the SEM chamber while mounted on a holder; (3) the equipment is configured with the proper detector and electron beam parameters suited to the kind of analysis being conducted; and (4) the sample surface is scanned by an electron beam and the signals released are then recognized and utilized to create a picture. The image’s surface morphology, particle size and shape, and elemental makeup were all further examined. Analysis using a scanning electron microscope (SEM) at a constant applied voltage of 16 kV was performed to find out how the surface morphology of pure PVDF and ZnO affected the microstructure of PVDF films. As demonstrated in Figure 4a,b, the pristine PVDF membrane’s surface morphology is noticeably smoother than that of the PVDF polymer matrix that has contained nanofiller ZnO. PVDF film surface is porous compared to PVDF/ZnO film. The top view microscopic image shows the altered surface with increased roughness credited to the PVDF matrix’s inclusion of ZnO nanofiller.

Powerful analytical methods such as energy-dispersive spectroscopy (EDS) were used to identify the elements present in a sample. By blasting the sample with high-energy electrons, which causes the emission of distinctive X-rays, the EDS spectrum is acquired. These X-rays’ energy are measured and utilized to determine the elements that are present in the sample. The strength of the peaks in the EDS spectrum indicates the relative quantity of each element in the sample and corresponds to the distinctive X-ray peaks of the elements present in the sample. The elemental makeup of materials and the presence of trace elements or contaminants in our sample were both identified using the EDS spectrum. To conform the composite formation of the prepared films, the energy-dispersive spectroscopy (EDS) spectrum is also provided in Figure 4c,d, where it shows uniform distribution of the proper concentration based on composite mixture. The composition of PVDF and PVDF/ZnO is formed reflecting individual particle ratios, according to the EDS spectra. To produce nanocomposite, which constitutes spin coated films with a consistent structure and 15% ZnO, the optimal production parameters have to be met.

### 4.2. X-ray Diffraction (XRD) Analysis

**Figure 5:**
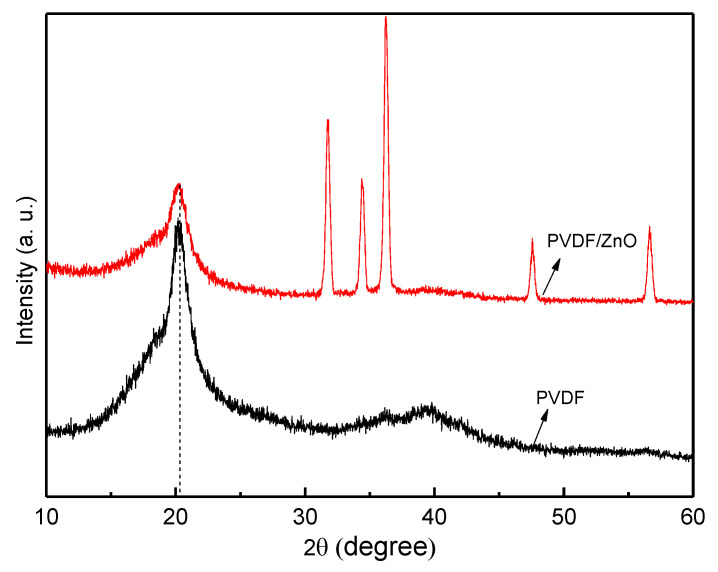
X-ray diffraction pattern of spin coated PVDF/ZnO composite structure.

X-ray diffraction (XRD) examination is a technique that examines the structure and characteristics of the materials by examining the crystallographic structures of the materials. The method entails illuminating the sample with X-rays and analyzing the ensuing diffraction pattern; we then utilized this pattern to ascertain the crystal structure and composition of the material. As X-rays are focused on a sample during XRD examination, they interact with the atoms in the crystal structure and scatter in various ways. The crystal structure and makeup of the substance were ascertained by analyzing the diffraction pattern created by the scattered X-rays on a detector. The results of an XRD pattern demonstrate the creation of a highly electroactive β-phase crystal structure and the presence of nanoparticles in the nanocomposite. The XRD signal at angle 2θ = 20.3° denotes β-phase development in nanocomposite [51]. The optimum sample’s (SN-8) XRD data are shown in Figure 5. The distinctive diffraction peaks of PVDF/ZnO are also present at 2θ = 31.78°, 34.35°, 36.26°, 47.71°, and 56.74°, which corresponds to the crystallinity characteristics of ZnO. The doping of ZnO in PVDF shows more crystallinity nature. Therefore, by adding ZnO particles, spin coating procedures might be used to create nanocomposite materials with increased piezoelectric characteristics and β-phase crystalline structure. Additionally, compared to the PVDF matrix, the piezoelectric characteristics of the piezoceramic ZnO particles are superior [52].

### 4.3. Fourier Transform Infrared Spectroscopy (FT-IR) Analysis

**Figure 6:**
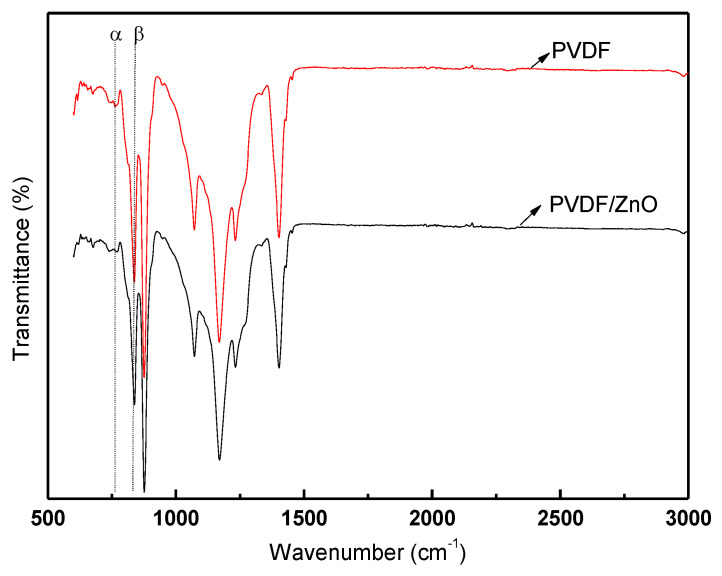
FTIR spectra of PVDF and PVDF/ZnO.

We used Fourier transform infrared spectroscopy (FT-IR) analysis to examine the sample’s infrared spectrum; this method recognizes and describes the chemical bonds and functional groups present in the sample. The method entails passing an infrared beam through the sample while measuring the beam’s transmission or absorption at various wavenumbers. The sample was initially put in the direction of the infrared beam during the FT-IR examination; the beam was then guided through the sample. Some of the infrared light was absorbed by the sample as the beam passed through it, which made certain chemical bonds vibrate. After that, the residual infrared light passed through the sample and was picked up by a detector. Information on the chemical bonds and functional groups present in the sample is available in the infrared spectrum results. Through an absorption band in the spectrum attributed to the absorption of infrared radiation by molecular vibration with a particular bond length and orientation, the FT-IR technique offers significant insight into the molecular bonding environment in a material. One of the most effective methods for studying the bonding environment in the polymer matrix and identifying various crystalline phases for PVDF polymer is FT-IR (see Figure 6).

With the loading of a small amount of the nanofillers, no appreciable changes in the structural and bonding environment of PVDF are seen. All composite membranes’ FTIR spectra contain the typical absorption bands that characterize the structural properties of PVDF. The distinctive absorption bands of the nonpolar α are seen at 763 cm^−1^, 795 cm^−1^, 855 cm^−1^, and 976 cm^−1^ in the FT-IR spectra for the pure PVDF film, while the bands of the polar β-phase are shown at 840 cm^−1^, 1276 cm^−1^, and 1431 cm^−1^, according to related reports in the literature [51,52,53,54]. By comparing the absorption peaks of the β and α phases at the wavelengths 840 cm^−1^ and 763 cm^−1^, respectively, it is possible to estimate the proportion of the beta crystalline phase in each sample. Using the Lambert-Beer law, the electroactive β-crystal content (F(β)) of the samples are evaluated [55].

$$F\left(\beta \right)=\frac{A_{\beta }}{\left(\frac{K_{\beta }}{K_{\alpha }}\right)A_{\alpha }+A_{\beta }}\times 100\%$$

**Figure 7:**
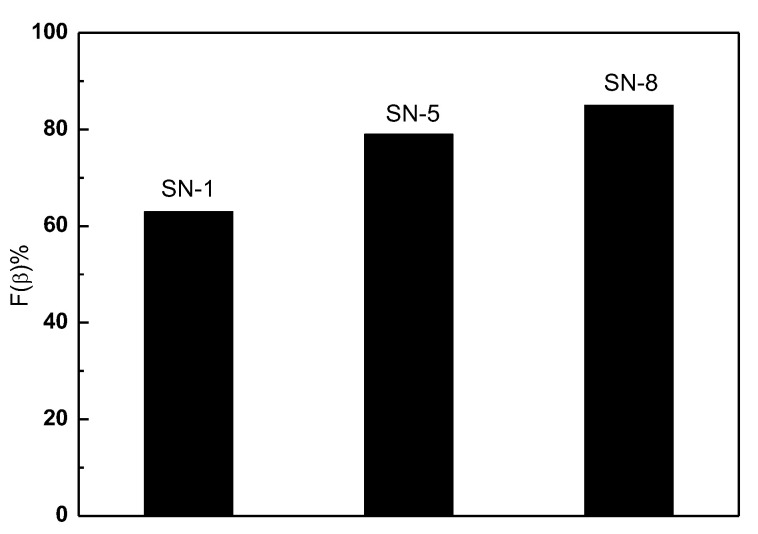
Electroactive β-crystal content, F(β).

Here, A_α_ and A_β_ is the absorbance of α and β phases at 763 cm^−1^ and 840 cm^−1^, respectively. K_α_ and K_β_ stand for the absorption coefficients, with respective values of 6.1 × 10^4^ and 7.7 × 10^4^ cm^2^mol^−1^. Figure 7 shows a bar chart representation of the evaluated F(β). It is found that the F(β) of PVDF nanofibers can be enhanced by adding ZnO nanoparticles. In addition, the PVDF/ZnO (SN-8) nanocomposite film exhibits a higher F(β) value (85%) than the only PVDF film (63%). The surface of nanoparticles with a negative charge density and the CH_2_ groups of the PVDF chains with a positive charge density effectively interact to cause the nucleation of the β-phase crystallite in nanoparticles doped PVDF composite [55,56].

### 4.4. Voltage Output

**Figure 8:**
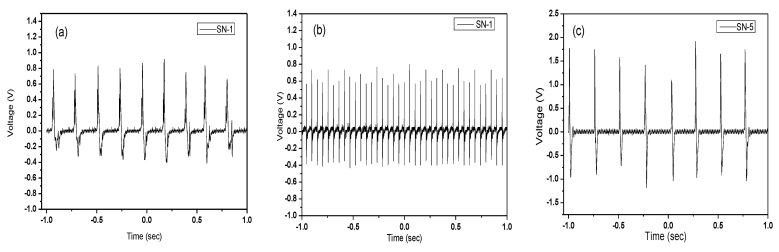

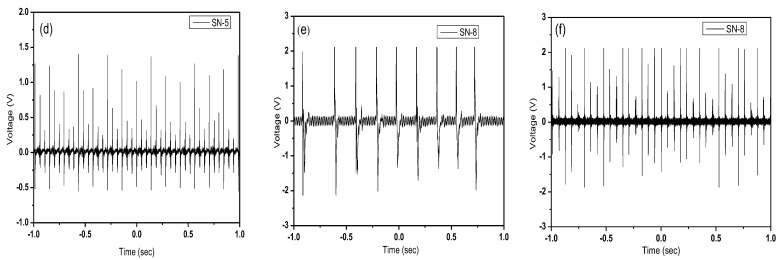
Open-circuit voltage under continuous hand pressing (**a**) PVDF-SN-1, (**c**) PVDF/ZnO-SN-5, (**e**) PVDF/ZnO-SN-8. Open-circuit voltage under continuous hitting (**b**) PVDF-SN-1, (**d**) PVDF/ZnO-SN-5, (**f**) PVDF/ZnO-SN-8.

**Figure 9:**
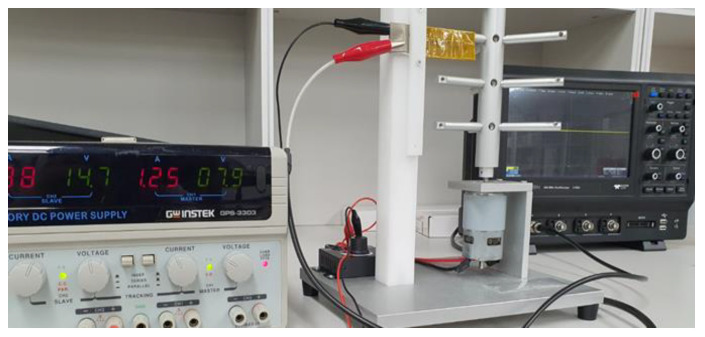
A picture of hitting machine setup.

The most crucial step in comparing the prepared sample’s piezoelectric capabilities is evaluating its electrical properties. The open-circuit voltage of our samples was tested for two different changeable conditions (hand press and hitting machine). The mechanical-to-electrical energy harvesting is seen in every situation and summarized in Table 2. The open-circuit voltage waveshapes from the oscilloscope are provided in Figure 8. Under continuous hand pressing on the sample, open-circuit voltage is seen in the oscilloscope in the case of hand pressing. It shows that PVDF/ZnO film (SN-8) produces a higher voltage (4.2 V) than pristine PVDF (1.2 V). In the case of continuous hitting with a hitting machine, the open-circuit voltage of the PVDF/ZnO film (SN-8) is also higher (3.9 V) than PVDF (1.1 V). A specially built hitting machine was utilized in the continuous hitting machine method, connected to a dc supply to turn the motor and an oscilloscope to measure the voltage output from the PNG (see Figure 9). The electrical response of the films increases with the increase in ZnO nanoparticles, as previously demonstrated. In this instance, the open-circuit voltage of the PVDF/ZnO nanocomposite film is over 3.5 times greater than that of PVDF films.

## 5. Conclusions

In this work, poly (vinylidene fluoride) and ZnO composites were used to spin-coat piezoelectric nanogenerators. The amount of ZnO in the composite, the amount of mechanical force used, and coatings were discovered to be some of the variables that affected how much power the nanogenerators produced. Different percentages of uniform nanofiber composites made of zinc oxide and polyvinylidene fluoride were synthesized. To achieve this synthesis, a 26% (wt./wt.) polymer solution was created under the stated conditions and the specified weights of zinc oxide were then added. Thin films were generated using a modified process that included 15 wt.% of ZnO and at various percentages. According to XRD, FT-IR, and SEM analysis, adding nanoparticles improved the crystal structure of manufactured devices. Additionally, the electrical response of samples based on consistently applied mechanical pressures demonstrated that the inclusion of nanoparticles improved the electrical output significantly. Finally, it might be said that the creation of nanocomposite devices would boost profit from each component’s existence and improve the device’s performance. This nanocomposite structure may offer a straightforward, practical, affordable, and versatile method for creating self-powering microelectronic devices for a variety of applications.

## Figures and Tables

**Table 1:** Sample fabrication summary used in this work.

Sample No.	PVDF (wt.%)	ZnO (wt.%)
SN-1	26	0
SN-2	26	2
SN-3	26	4
SN-4	26	6
SN-5	26	8
SN-6	26	10
SN-7	26	12
SN-8	26	15

**Table 2:** Electrical response of prepared PNGs.

Measuring Method	Sample No.	ZnO (wt. %)	Voltage (V)	Improvement (%)
Hand press	SN-1	0	1.21	-
	SN-5	8	2.63	117
	SN-8	15	4.22	60
Hitting machine	SN-1	0	1.13	-
	SN-5	8	1.96	73
	SN-8	15	3.94	101

## Data Availability

The data created in this study are fully depicted in the article.
